# Deferoxamine attenuates lipopolysaccharide-induced neuroinflammation and memory impairment in mice

**DOI:** 10.1186/s12974-015-0238-3

**Published:** 2015-02-03

**Authors:** Xiao-Ying Zhang, Jiang-Bei Cao, Li-Ming Zhang, Yun-Feng Li, Wei-Dong Mi

**Affiliations:** Anesthesia and Operation Center, Chinese PLA General Hospital, Beijing, 100853 China; Institute of Pharmacology and Toxicology, Academy of Military Medical Sciences, Beijing, 100850 China

**Keywords:** Deferoxamine, Neuroinflammation, Iron, Memory impairment, Oxidative stress, Apoptosis

## Abstract

**Background:**

Neuroinflammation often results in enduring cognitive impairment and is a risk factor for postoperative cognitive dysfunction. There are currently no effective treatments for infection-induced cognitive impairment. Previous studies have shown that the iron chelator deferoxamine (DFO) can increase the resistance of neurons to injury and disease by stimulating adaptive cellular stress responses. However, the impact of DFO on the cognitive sequelae of neuroinflammation is unknown.

**Methods:**

A mouse model of lipopolysaccharide (LPS)-induced cognitive impairment was established to evaluate the neuroprotective effects of DFO against LPS-induced memory deficits and neuroinflammation. Adult C57BL/6 mice were treated with 0.5 μg of DFO 3 days prior to intracerebroventricular microinjection of 2 μg of LPS. Cognitive function was assessed using a Morris water maze from post-injection days 1 to 3. Animal behavioral tests, as well as pathological and biochemical assays were performed to evaluate the LPS-induced hippocampal damage and the neuroprotective effect of DFO.

**Results:**

Treatment of mice with LPS resulted in deficits in cognitive performance in the Morris water maze without changing locomotor activity, which were ameliorated by pretreatment with DFO. DFO prevented LPS-induced microglial activation and elevations of IL-1β and TNF-α levels in the hippocampus. Moreover, DFO attenuated elevated expression of caspase-3, modulated GSK3β activity, and prevented LPS-induced increases of MDA and SOD levels in the hippocampus. DFO also significantly blocked LPS-induced iron accumulation and altered expression of proteins related to iron metabolism in the hippocampus.

**Conclusions:**

Our results suggest that DFO may possess a neuroprotective effect against LPS-induced neuroinflammation and cognitive deficits via mechanisms involving maintenance of less brain iron, prevention of neuroinflammation, and alleviation of oxidative stress and apoptosis.

## Background

Neuroinflammation has been reported as a part of the neuropathogenesis of cognitive impairment [1]. The elderly are vulnerable to the adverse effects of infections on cognitive function, and the aging process itself is associated with enhanced neuroinflammatory processes involving microglial activation and production of pro-inflammatory cytokines [2,3]. Neuroinflammation also occurs in association with the pathological changes in the brain of patients who have undergone an operation and patients with Alzheimer’s disease (AD) or ischemic stroke [4,5]. However, the exact mechanism underlying the effect of neuroinflammation on cognitive function has not been completely clarified yet.

Lipopolysaccharide (LPS) is a major bacterial TLR4 ligand that activates the innate immune response to infections, and administration of LPS by systemic injection [6], intracerebral microinjection, or chronic infusion [7-9] can cause cognitive impairment in animal models. Previous studies have shown that LPS can result in cognitive impairment through mechanisms involving expression of pro-inflammatory cytokines and neuronal death via apoptosis [10-14]. It is known that pro-inflammatory mediators disrupt hippocampal neuronal functions, including long-term potentiation and working memory consolidation [15,16]. Cytokines such as TNF-α and IL-1β are involved in hippocampal long-term potentiation and dendritic branching, which are processes involved in memory formation and maintenance [17]. Activation of microglia by LPS has been linked to the pathogenesis of neuronal death, neurogenesis failure, and hippocampus-dependent memory and synaptic plasticity impairments; however, the mechanisms responsible for these effects are not well understood [18], although brain tissue oxidative damage has been considered an important contributor to memory impairment induced by LPS [19].

Evidence also suggests that aberrant iron accumulation in the brain plays a pivotal role in the pathogenesis of many diseases involving cognitive dysfunction [20-22]. The major mechanism of iron-mismanagement was related to increased iron uptake by DMT1, Tf/TfR2, and Lf/LfR [23-25], decreased iron export by FPN and Cp [26,27], and iron storage misregulation by ferritin and lysosomes [28-30]. In fact, it has been demonstrated that iron overload can lead to free radical formation, oxidative stress, and neuronal damage [20,31-34]. Neuroinflammation has been found in many iron-associated neurodegenerative diseases such as AD, Parkinson’s disease (PD), and demyelinating diseases such as multiple sclerosis and amyotrophic later sclerosis [35-39].

Based on the above findings, we hypothesized that decreasing iron content in the brain during neuroinflammation might decrease microglial activation and inflammatory cytokines in the hippocampus, and thus improve cognitive impairment. To test this hypothesis, we assessed the neuroprotective effects of iron chelator deferoxamine (DFO) against LPS-induced neuroinflammation in the present study. In order to remove any potential confounding effects of the peripheral immune system, the present set of experiments were carried out by challenging mice by intracerebroventricular injection of LPS. The results obtained in this study may provide new insights into the potential novel mechanisms for the treatment of cognitive impairment.

## Methods

### Animals

Male C57BL/6 mice aged 10 to 12 weeks, weighing 20 to 22 g, were obtained from the Beijing SPF Animal Technology Company (Beijing, China). The animals were housed in a temperature- and humidity-controlled room with a 12-h light/dark cycle starting at least 5 days before the experiment and had access to water and food *ad libitum*. They were group-housed with the same mates throughout the acclimation and testing periods. Animal experiments were performed in compliance with the current laws of China and the National Institutes of Health Guide for the Care and Use of Laboratory Animals (NIH publication No. 86–23, revised 1996).

### Establishment of a mouse model of LPS-induced cognitive impairment

To optimize the dose of LPS for inducing cognitive impairment, mice were randomly assigned into five groups (*n* = 8 for each) and administered with 0, 0.01, 0.1, 2, and 5 μg of LPS (in 2 μL of artificial cerebrospinal fluid (aCSF); Sigma, St. Louis, MO, USA) by stereotactic intracerebroventricular injection 5 days after acquisition training in a Morris water maze (MWM). The aCSF vehicle contained 140 mM NaCl, 3.0 mM KCl, 2.5 mM CaCl_2_, 1.0 mM MgCl_2_, and 1.2 mM Na_2_HPO_4_, adjusted to pH 7.4. The probe test for reference memory was conducted 1 day after LPS administration, and working memory was tested on days 1 to 3 after LPS administration to observe whether the impairment could recover spontaneously and the duration it needed.

### Optimization of dose of DFO to alter LPS-induced cognitive impairment

Six randomly assigned groups of mice were intracerebroventricularly administrated with 0, 0, 0.1, 0.5, 2.5, and 5 μg of DFO (in 2 μL of aCSF; Sigma) 3 days prior to microinjection of LPS. All groups received intracerebroventricular administration of LPS (2 μg in 2 μL of aCSF), except the first group that received equal volume of aCSF and served as a control group. Mice were allowed to rest for 6 h before MWM acquisition training on the day of DFO administration and the probe test for reference memory was conducted 1 day after LPS administration.

### Effect of DFO on LPS-induced cognitive impairment in mice

One hundred mice were randomly assigned into four groups: control, DFO, LPS, and LPS + DFO (*n* = 25 for each). Intracerebroventricular administration of DFO (0.5 μg in 2 μL of aCSF) was commenced 3 days prior to microinjection of LPS (2 μg in 2 μL of aCSF), while the control group received equal volume of aCSF.

For stereotactic injection of LPS and DFO, mice were anesthetized with Avertin (200 mg/kg, intraperitoneal injection) and placed on a stereotactic apparatus (Kopf Instruments, Tujunga, CA, USA). Injection was performed through drilled holes in the skull, into the paracele using the following coordinate (in mm): 0.5 posterior, ± 1.0 lateral and 2.0 ventral from bregma. The injection speed was set at 0.667 μL/min and the needle was left in place for 1 min following injection.

Body weights were determined daily. The mice were sacrificed by CO_2_ asphyxiation 6, 24, 48, or 72 h following administration of LPS, followed by transcardial perfusion with ice-cold PBS. The brain of five mice killed at 24 h in each group were immediately removed, fixed in 4% paraformaldehyde for 48 h and cryoprotected in 30% sucrose for 48 h at 4°C, followed by histological analysis. The hippocampus of the other mice were rapidly dissected out and stored at −80°C until analysis. Tissues of mice killed at 6 h were used for enzyme-linked immunosorbent assay (ELISA) for measuring inflammatory cytokines because it is known that LPS activates microglia and consequently induces pro-inflammatory protein secretion within 6 h in the mouse hippocampus via the NF-κB pathway [40,41]. All other tests were carried out on postinjection day 1 that corresponded to the time point of the peak of behavioral deficits.

### Open field test

To evaluate whether the reversion of lesioned performance by LPS depends on altering the locomotor activity, we assessed the numbers of line crossings and rears in mice [42]. Mice were placed in the corner of a plastic box (36 × 29 × 23 cm) in which the base was divided into equal sectors for a 5-min acclimation period, and then the numbers of crossings (with all four paws placed into a new square) and rears (with both front paws raised from the floor) were recorded over the next 5 min. The open field was cleaned with 5% ethyl alcohol and allowed to dry between tests.

### MWM test

The MWM test, which is a hippocampal-dependent test of spatial learning and memory for rodents, was performed as described previously with minor modifications [43]. In this test mice rely on distal cues to navigate from start locations around the perimeter of an open swimming arena (diameter, 122 cm; water temperature, 22°C) to locate a submerged escape platform (10 cm^2^). Spatial learning is assessed across repeated trials for 5 days (day −5 to day −1). The pool was situated in a room with visual cues. The animals’ movements were recorded with a video camera attached to the ceiling. Mice were released into the water facing the wall of the pool from one of four separate quadrants. In all the trials, mice were allowed to swim until they landed on the platform. If a mouse failed to find the platform within 60 s, it was picked up and placed on the platform for 10 s. After that, the mouse was removed to its cage and the second animal was tested on Trial 1. This rotation was repeated until all animals completed Trial 1. Subsequently, the process was repeated for subsequent trials until four trials completed per day for 5 consecutive days. After the daily session, each mouse was dried under a heater and returned to the home cage. On the third day of acquisition training (day −3), microinjection of DFO (or ACSF) was executed 6 h before training. On day 0, animals underwent LPS microinjection. On postoperative day 1, mice were subjected to the probe test for reference memory during which the platform was absent. Swimming speed, platform-site crossings, distance around the platform, and the percentage of distance travelled in the target quadrant were recorded. Each mouse was placed in the pool once for 60 s, starting from the opposite quadrant to the platform. Reference memory was determined by preference for the platform area. On days 1, 2 and 3, working memory was tested, during which both the platform and mice were randomly placed in a novel position to assess working- or trial-dependent learning and memory. In this procedure, which is also called matching-to-sample, the animal is given two trials per day. On each day, the first trial represents a sample trial. During the sample trial, the animal must learn the new location of the platform by trial and error. Trial 2 is the test or matching trial in which savings in recall between Trial 1 and Trial 2 are measured. Trial 2 begins after a 15-s inter-trial interval. If the animal recalls the sample trial, it will swim a shorter path to the goal on the second trial. As the platform is moved daily, no learning of platform position from the previous day can be transferred to the next day’s problem; hence, recall on each day during Trial 2 is dependent on that day’s sample trial and measures only temporary or working memory, during which the latency to the novel platform was recorded.

### Immunofluorescence staining

Coronal sections (12 μm) were cut through the entire hippocampus using a Microm HM550 cryostat (Germany). Immunofluorescence staining was performed as previously described by Jennifer *et al*. [44]. Briefly, sections were incubated in 0.05% H_2_O_2_ in 0.1 M PBS for 20 min to block endogenous peroxidase, and in 2% goat serum/0.1% Triton X-100 in 0.1 M PBS for 1 h to block non-specific binding sites. The sections were then incubated with the primary antibody (rabbit anti-Iba1, 1:500; Wako) to label microglia at 4°C overnight. Following that, the sections were incubated with the appropriate secondary antibody (anti-rabbit IgG, 1:200; Jackson, USA) for 2 h at room temperature. Glial reactivity is characterized by an increase in the number of cells and an alteration in cell morphology (rounding of the cell bodies and thickening of processes), which lead to an increase in labeling with increasing glial reactivity. An increase in the integrated intensity/pixel area for Iba1 (ionized calcium-binding adaptor molecule 1) staining was interpreted to signify microglial reactivity. The number of Iba1-labeled cells per view was counted using fluorescence microscopy at × 20 magnification and the mean density at × 100 magnification. Images were captured using the Leica TCS SP5 confocal imaging system and quantified using Image-Pro Plus 6.0 software.

### ELISA

Concentrations of IL-1β and TNF-α were examined by ELISA using IL-1β and TNF-α ELISA assay kits (R&D Systems, USA). Hippocampal tissues were homogenized in RIPA lysis buffer (Applygen, China). Supernatant protein concentrations were determined after centrifugation at 12,000 g for 15 min with a BCA protein assay kit (Pierce, USA). For each sample, 10 μg of extracted protein was used for detection. The procedure followed the manufacturer’s instructions. The absorbance was read on a spectrophotometer at a wavelength of 450 nm and a reference wavelength of 650 nm. The concentrations of IL-1β and TNF-α were calculated according to the standard curve and presented as pg/μg protein.

### Western blot analysis

Western blot was performed following the manufacturer’s instructions. Equal amounts (50 μg) of proteins were separated by SDS-PAGE and analyzed by western blot using the following primary antibodies: anti-caspase-3 (1:500; Proteintech), anti-pGSK-3β (Ser9) (1:1,000; Cell Signaling), anti-GSK-3β pan (1:1,000; Cell Signaling), anti-ferritin (Fn; 1:500; Abcam), and anti-ferroportin 1(FPN; 1:1,000; Lifespan). The expression of a housekeeping protein, β-actin, was measured using an anti-β-actin antibody (1:500; Santa Cruz). Each experiment was repeated at least four times. Relative expression levels of proteins were normalized to β-actin.

### Iron content determination

Total iron content in the hippocampus was determined using a flame atomic absorption spectrophotometer (VarianAA240FS, USA). Hippocampal tissues were weighed, dried at 65°C for 24 h and then digested at 100°C with nitric acid and 30% hydrogen peroxide [45]. Digested samples were well mixed and further diluted. A blank sample was included as a baseline reference for every run. Accuracy was checked using an internally prepared solution. The standard addition method was used for calibration. Standard and control samples were prepared in an identical manner to the experimental samples.

### MDA concentration and SOD activity assays

Level of malondialdehyde (MDA), a well-established indicator of lipid peroxidation, and the activity of superoxide dismutase (SOD), an endogenous scavenger of reactive oxygen species (ROS), in the hippocampal tissue were measured using commercial assay kits (Nanjing Jiancheng, China) according to the manufacturer’s instructions. The results of MDA and SOD are expressed as nanomoles of MDA per milligram of total protein (nmol/mg protein) and units per milligram of total protein (U/mg protein), respectively.

### Statistical analysis

All data were analyzed by an observer who was blind to the experimental protocol. Intergroup comparisons were conducted by two-way analysis of variance (2 × 2 ANOVA), followed by a Dunnett post hoc test where necessary. For acquisition training (days −1 to −5) and spatial working memory testing (days 1 to 3), data were analyzed using two-way ANOVA (treatment × trial time) with repeated measures (trial days) followed by the Bonferroni post hoc test. For all other data, two-way ANOVA was used. All results are expressed as mean ± standard error (SE), and *P* values <0.05 were considered statistically significant.

## Results

### Deferoxamine prevents memory deficits caused by experimental cerebral inflammation

**Figure 1:**
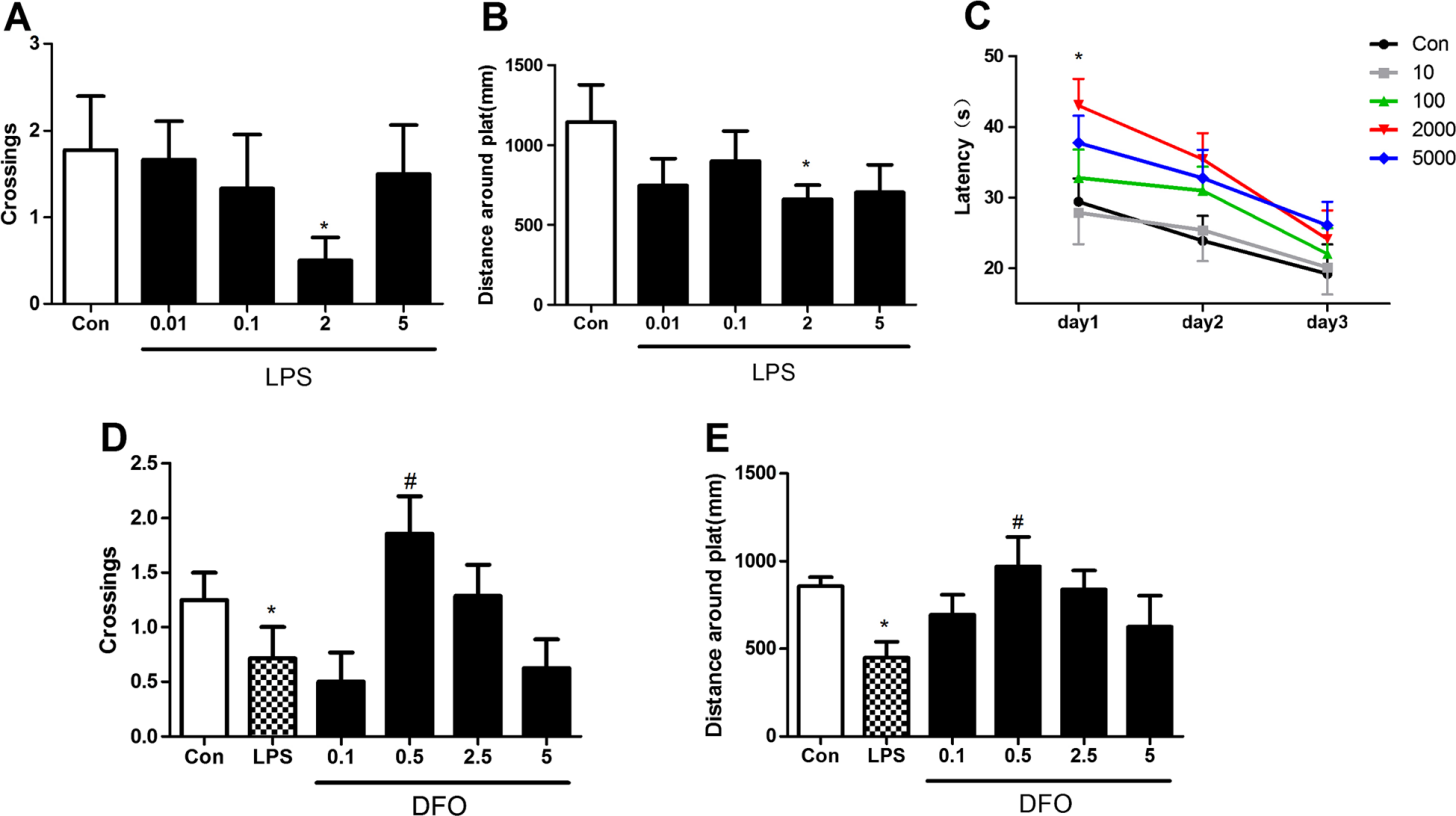
**Preliminary experiments to test the optimal doses of LPS to induce cognitive dysfunction and DFO to attenuate it by the Morris water maze test.** Intracerebroventricular LPS reduced platform crossings **(A)** and distance around the plat **(B)** in reference memory test 1 day following microinjection after 5 days of training, and latency to plat **(C)** in working memory on post-injection days 1 to 3, 2 μg LPS in particular. DFO attenuated LPS-induced reduction of crossings **(D)** and distance around the platform **(E)**, with 0.5 μg of DFO being more significant. Data are expressed as mean ± SE (*n* = 8). *P <0.05 *vs*. control; #*P* <0.05 *vs*. LPS. Con, control; DFO, deferoxamine; LPS, lipopolysaccharide; SE, standard error.

To elucidate the effect of LPS and DFO on hippocampal-dependent learning and memory, an MWM test was conducted. In the preliminary experiment designed for determining the optimal dose of LPS, intracerebroventricular administration of 2 μg of LPS (in 2 μL of aCSF) induced memory impairment but no swimming speed change in the MWM. Mice in the LPS group performed worse on day 1 (*P* = 0.0449 for crossings and *P* = 0.0406 for distance around the platform in reference memory test; *P* =0.0162 for latency in working memory test; *n* = 8; Figure 1A-C) following microinjection after 5 days of acquisition training, but the performance returned to control levels on day 3, which suggested that LPS induced a temporary impairment in spatial learning and memory (*P* = 0.2232 for latency in working memory test; *n* = 8; Figure 1C).

On above basis, the experiment for determining the optimal dose of DFO showed that intracerebroventricular administration of 0.5 μg of DFO (in 2 μL of aCSF) 3 days before LPS microinjection could ameliorate the memory deficits (*P* = 0.0122 for crossings; *P* = 0.0215 for distance around the platform; *n* = 8; Figure 1D, E).

**Figure 2:**
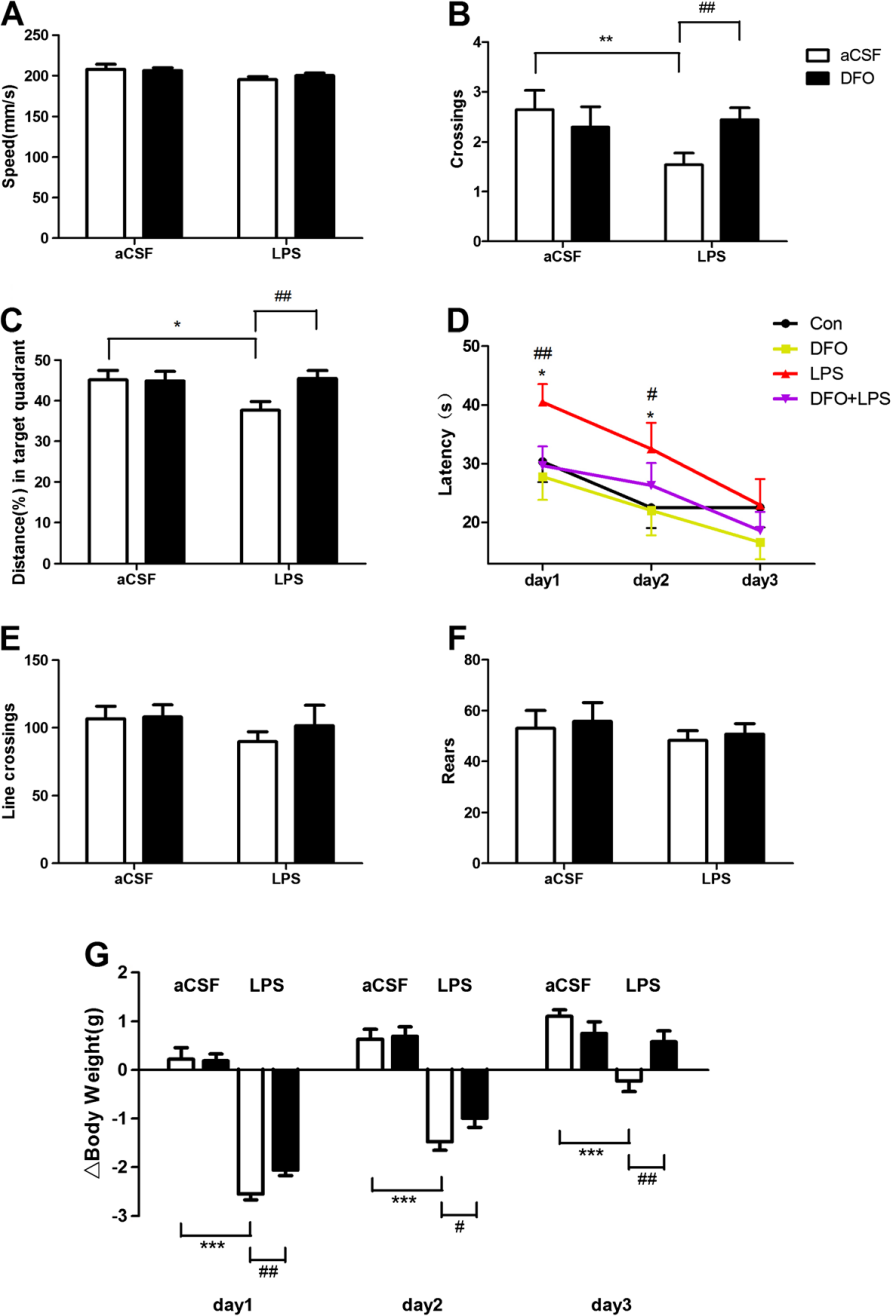
**DFO ameliorates behavioral performance in LPS-exposed mice.** The Morris water maze test was performed as described in the Methods section. **(A)** Swimming speed during probe testing. **(B)** Platform-site crossings during probe testing. **(C)** Percentage of distance travelled in the target quadrant during probe testing. **(D)** Latency to the platform during spatial working memory testing on days 1, 2, and 3. **(E, F)** Results of spontaneous locomotor activity showing that neither LPS nor DFO altered the number of line crossings **(E)** or rears **(F)** 24 h after LPS administration. **(G)** Changes of body weight on days 1, 2, and 3. Data are expressed as mean ± SE (*n* = 25). *P <0.05, **P <0.01, ***P <0.001 *vs*. control; #*P* <0.05, ##*P* <0.01 *vs*. LPS. Con, control; DFO, deferoxamine; LPS, lipopolysaccharide; SE, standard error.

To elucidate the effect of LPS and DFO on hippocampal-dependent learning and memory, an MWM test was conducted [43]. Mice initially received 5 days of acquisition training with the platform in a fixed location. On day 6, animals underwent microinjection surgery, and on postoperative day 1, mice were subjected to a probe test for reference memory, during which the platform was absent, as well as an open field test for locomotion activity. On days 1 to 3, mice were subjected to a working memory test, during which both the platform and mice were randomly placed in a novel position to assess working- or trial-dependent learning and memory. If the animal recalls the sample trial, it will swim a shorter path to the goal on the second trial. Two-way ANOVA revealed that during the acquisition phase, the performance of all groups improved over time, and no differences were observed between groups (data not shown). During the probe test, there were no significant differences in swimming speed between groups, suggesting that the poorer performance of the LPS group was not a result of reduced motor ability (Figure 2A). It was observed that deferoxamine prevented memory deficits caused by LPS on postoperative day 1 (*F*_LPS_ = 2.441, *F*_DFO_ = 0.8651, *F*_LPS×DFO_ = 4.186 for platform-site crossings, *P* <0.05; *F*_LPS_ = 2.192, *F*_DFO_ = 2.693, *F*_LPS×DFO_ = 3.070 for percentage of distance travelled in the target quadrant during probe testing, *P* <0.05; Figure 2B, C). Similar results were observed in latency to the platform during the working memory test and performance returned to control levels on day 3 (*F*_LPS_ = 2.924 and 3.078, *F*_DFO_ = 3.652 and 0.6747, *F*_LPS×DFO_ = 1.417 and 0.4951 for days 1 and 2, respectively, *P* <0.05; Figure 2D).

To evaluate whether the lesioned performance caused by LPS depends on altering the locomotor activity, the open field test was conducted [46]. No significant difference was observed in the locomotor performance (line crossings and rears) 24 h after LPS treatment (LPS and DFO + LPS groups) when compared with the control and DFO groups (Figure 2E, F).

To confirm that central LPS induced a sickness response, we measured the change in body weight [47,48]. As expected, mice intracerebroventricularly injected with 2 μg of LPS lost body weight over a 3-day period following treatment (*F*_LPS_ = 290.9, 95.34, and 9.024 for days 1, 2, and 3, respectively, *P* <0.05; Figure 2G). However, that loss was significantly reduced by pretreatment with DFO (*F*_DFO_ = 2.427, 1.974, and 0.7966 for days 1, 2, and 3, respectively, *P* <0.05; Figure 2G).

Collectively, our results suggest that LPS causes cognitive impairment, specifically a deficit in short-term memory retention, which can be ameliorated by DFO pretreatment.

### DFO suppresses LPS-induced accumulation of inflammatory proteins in the hippocampus

**Figure 3:**
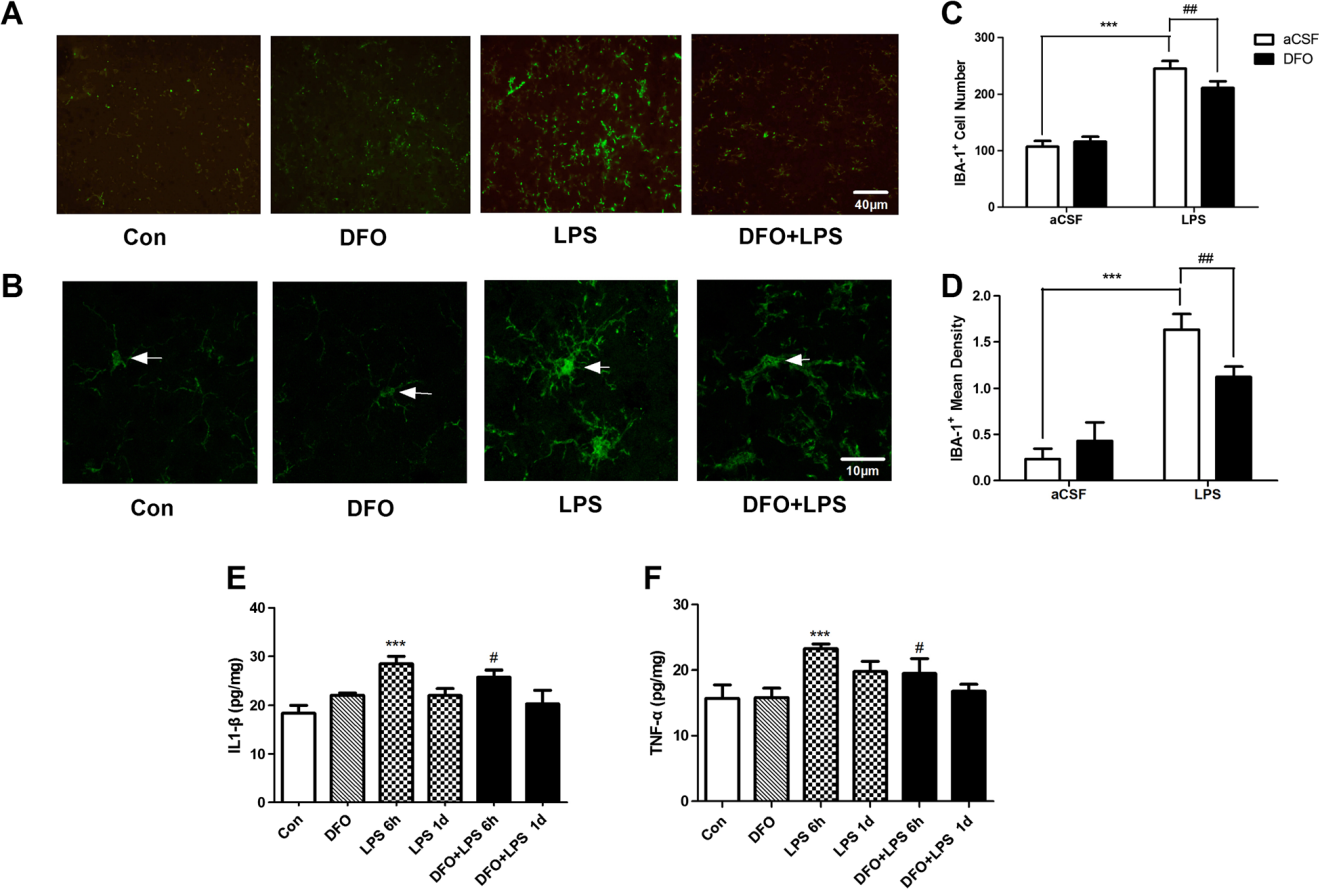
**Deferoxamine (DFO) attenuates lipopolysaccharide (LPS)-induced microglial activation and inflammatory cytokine accumulation. (A, B)** Representative images of Iba1-labeled activated microglia in the hippocampal C1 area. Activated microglia is shown in green. Scale bars = 40 μm for **(A)** and 10 μm for **(B)**. **(C, D)** Quantification of the Iba^+^ cell average number and the mean grey intensity. **(E, F)** Levels of IL-1β and TNF-α in samples of the hippocampus. Data are expressed as mean ± SE (*n* = 6). *P <0.05, ***P <0.001 *vs*. control; #*P* <0.05 *vs*. LPS. Con, control; DFO, deferoxamine; LPS, lipopolysaccharide; SE, standard error.

In view of the important role that cytokines and microglial activation play in LPS-induced neuroinflammation [8,11,49,50], we investigated the levels of several pro-inflammatory cytokines and proteins (IL-1β, TNF-α, Iba1) in the hippocampus, a brain region where neuroinflammation mainly occurs in response to brain injury and inflammation [51,52]. Immunohistochemistry and ELISA immunoassay showed that LPS caused obvious microglial activation labeled by Iba1 (*F*_LPS_ =105.3, *P* <0.001 and *F*_LPS_’ = 46.27, *P* <0.01 for number and density, respectively; Figure 3C, D) and significant increases in IL-1β (*t* = 0.0005 control *vs*. LPS 6 h, *n* = 6; Figure 3E) and TNF-α levels (*t* = 0.0006 control *vs*. LPS 6 h, *n* = 6; Figure 3F) in the hippocampus. Remarkably, DFO significantly attenuated LPS-induced microglial activation (*F*_DFO_ = 5.208, *P* <0.01 and *F*_DFO_’ = 3.549, *P* <0.01 for number and density, respectively; Figure 3C, D) in the hippocampus of mice, as well as increases in IL-1β (*t* = 0.0388 LPS 6 h *vs*. DFO + LPS 6 h, *n* = 6; Figure 3E) and TNF-α (*t* = 0.0.0411 LPS 6 h *vs*. DFO + LPS 6 h, *n* = 6; Figure 3F) levels.

### DFO pretreatment prevents caspase-3 increase and blocks glycogen synthase kinase 3β (GSK3β) activity in mice with LPS-induced hippocampal impairment

**Figure 4:**
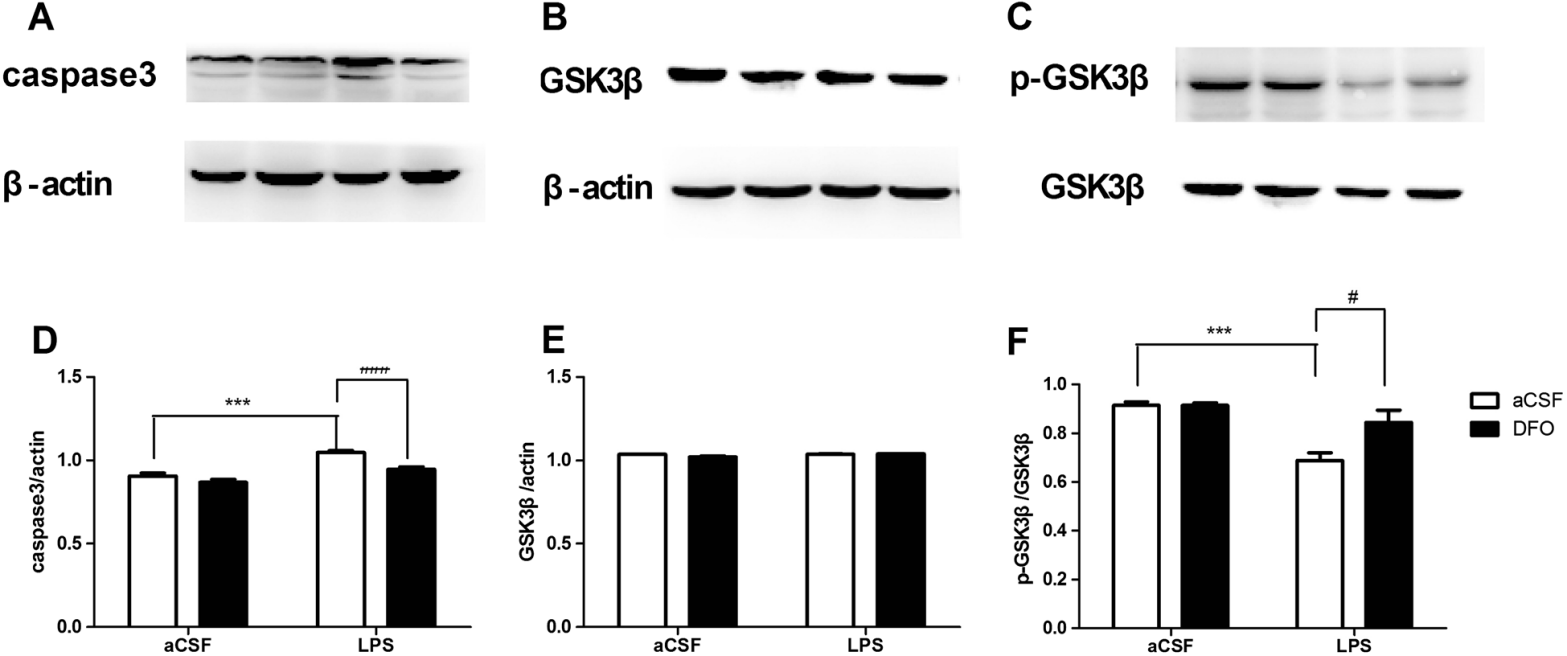
**Effect of DFO on the expression of caspase-3 and p-GSK3β/GSK3β proteins in mice with LPS-induced hippocampal impairment. (A-C)** Protein bands on gel and their relative intensities. **(D-F)** The expression levels of caspase-3 and p-GSK3β/GSK3β proteins were normalized to that of β-actin as an internal control. Data are expressed as mean ± SE (*n* = 4–5). *P <0.05, ***P <0.001 *vs*. control; #*P* <0.05, ###*P* <0.001 *vs*. LPS. Con, control; DFO, deferoxamine; LPS, lipopolysaccharide; SE, standard error.

To investigate cell death following intracerebroventricular administration of LPS with or without pretreatment of DFO, apoptotic marker caspase-3 was evaluated [53]. Western blot analysis showed that LPS induced caspase-3 activation; however, pretreatment with DFO attenuated caspase-3 activation (*F*_LPS_ = 50.93, *P* <0.001; *F*_DFO_ = 20.77, *P* <0.001; Figure 4D), suggesting that DFO may alleviate LPS-induced apoptosis in the hippocampus.

Given the critical roles of GSK3β in apoptosis and cognitive function, we extended our experiments to determine whether DFO is able to modulate GSK3β activity in the LPS-exposed mouse hippocampus [54,55]. The total expression level of GSK3β was not changed by different treatments (*F*_LPS_ = 5.67, *P* >0.05; *F*_DFO_ = 2.56, *P* >0.05; Figure 4E), whereas the phospho-GSK3β/GSK3β ratio in the hippocampus of LPS-lesioned mice was significantly decreased, and DFO attenuated this reduction (*F*_LPS_ = 23.36, *P* <0.001; *F*_DFO_ = 6.663, *P* <0.05; Figure 4F). These data suggest that DFO treatment downregulates GSK3β activity by phosphorylating GSK3β in the hippocampus. In the LPS-treated hippocampus, total GSK3β remained unchanged and the activation of GSK3β was relatively increased compared with the vehicle control. Collectively, our data revealed that LPS and DFO antagonistically exert their effects on caspase-3 accumulation and p-GSK3β/GSK3β pathways in the mouse hippocampus.

### DFO reverses altered iron content and expression of Fn and FPN proteins in the hippocampus of LPS-exposed mice

**Figure 5:**
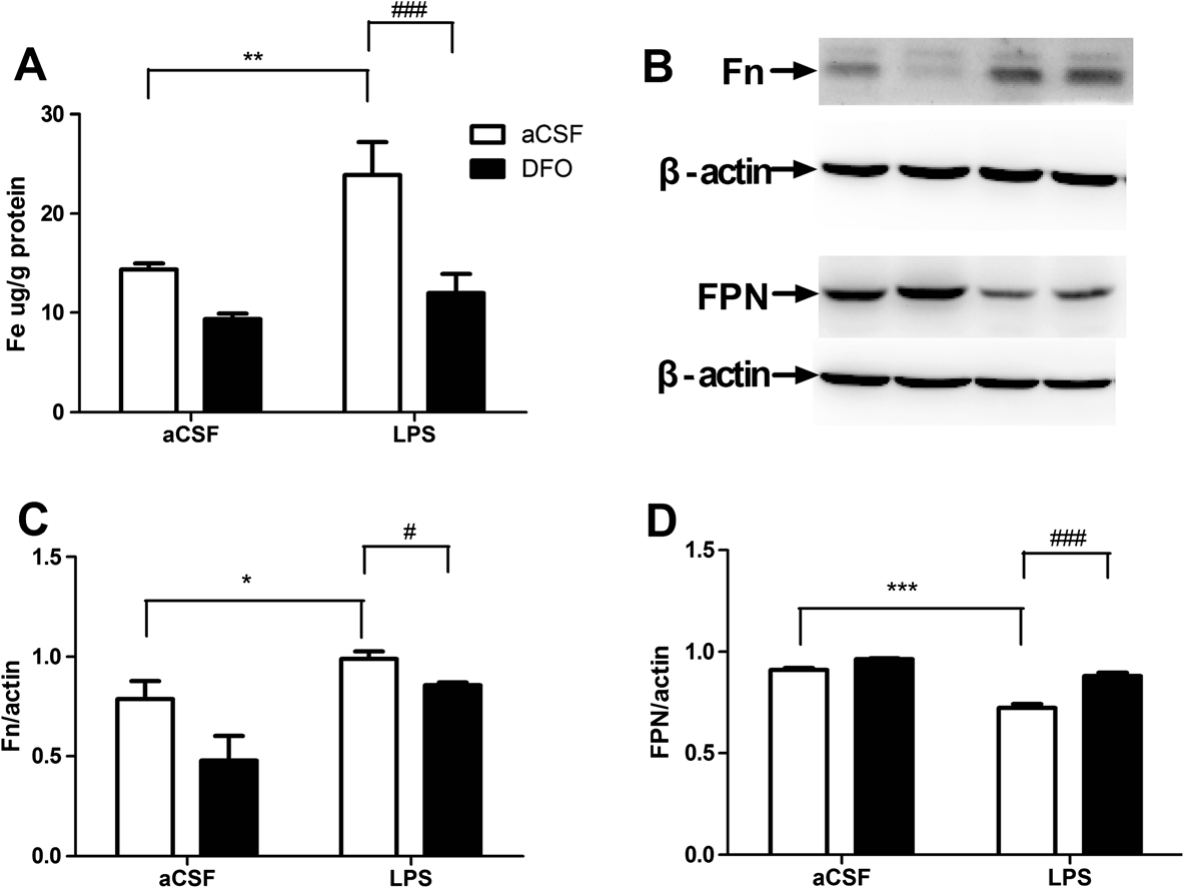
**Effect of DFO on iron content and expression of Fn and FPN proteins in mice with LPS-induced hippocampal impairment. (A)** Iron content in the hippocampus is indicated as μg per gram tissue (*n* = 8). **(B)** Protein bands on gel and their relative intensities. **(C, D)** The expression levels of Fn and FPN proteins were normalized to that of β-actin as an internal control in (*n* = 4–5). Data are expressed as mean ± SE. *P <0.05, **P <0.01, ***P <0.001 *vs*. control; #*P* <0.05, ###*P* <0.001 *vs*. LPS. Con, control; DFO, deferoxamine; LPS, lipopolysaccharide; SE, standard error.

To determine the changes in iron content and metabolism, we investigated the effect of LPS or DFO on iron content and the expression levels of proteins involved in the maintenance of brain iron homeostasis by flame atomic absorption spectrophotometery and western blot, respectively [20,45]. A significant increase in iron content was observed on postoperative day 1 following LPS microinjection in the LPS group, and the biggest increase was 66.16% as compared with the control group (*F*_LPS_ = 9.588, *P* <0.01; *n* = 8; Figure 5A). Nevertheless, DFO dramatically blocked this increase (*F*_DFO_ = 18.61, *P* <0.001; *n* = 8; Figure 5A). Fn is a ubiquitous and highly conserved iron-binding protein, and it is a major form of non-heme iron store in cells. The levels of Fn immunoreactivity increased compared with those in controls on postoperative day 1, and consistent with the hypothesis that DFO is a high-affinity iron chelator to bind Fe^3+^, especially Fn and heme iron, DFO reversed accumulation of Fn in the hippocampus of LPS-exposed mice (*F*_LPS_ = 13.42, *P* <0.05; *F*_DFO_ = 7.695, *P* <0.01; *n* = 5; Figure 5C). On the contrary, FPN, an iron exporter which is present extensively on the neurons, oligodendrocytes and astrocytes and plays an important and probably even only role in iron efflux from these cells, decreased in the LPS group, and the decrease was weakened by DFO pretreatment (*F*_LPS_ = 99.48, *P* <0.001; *F*_DFO_ = 60.82, *P* <0.001; *n* = 4; Figure 5D).

### DFO protects mice from LPS-induced increase of MDA level and decrease of SOD activity

Given that increased levels of iron can lead to oxidative stress, which in turn is detrimental to neuronal function [56,57], we assessed whether LPS was also linked to an increase in oxidative stress and whether improvement of poor behavior and above impairment was related to a decrease in oxidative stress in the hippocampus. We measured the level of MDA, which is a byproduct of lipid peroxidation and indicator of oxidative stress, and the activity of SOD, which is one of the major antioxidant enzymes involved in protecting the nervous tissue from oxidative stress. Our data indicated that MDA level was about twice that of the control and that SOD activity was reduced in the hippocampus of mice in the LPS group on postoperative day 1 (*F*_LPS_ = 22.05, *P* <0.001, *n* = 9 for MDA; *F*_LPS_ = 90.59, *P* <0.001, *n* = 12 for SOD; Table 1). However, the status of less iron, which DFO treatment resulted in, considerably weakened this lesion (*F*_DFO_ = 0.8509, *P* <0.05, *n* = 9 for MDA; *F*_DFO_ = 0.2774, *P* <0.05, *n* = 12 for SOD; Table 1). Meanwhile, no significant difference was found between the DFO-alone and control groups.

**Table 1:** **MDA concentration (nmol/mg protein) and SOD activity (U/mg protein).**

	**Con**	**DFO**	**LPS**	**DFO + LPS**
SOD	109.2 ± 4.971	98.11 ± 10.55	51.05 ± 6.99^a^	67.03 ± 5.18^b^
MDA	0.1752 ± 0.0366	0.216 ± 0.0303	0.362 ± 0.0124^a^	0.3346 ± 0.0115^b^

Data are expressed as mean ± SE.

^a^ *P* <0.001 *vs*. control.

^b^ *P* <0.05 *vs*. LPS.

Con, control; DFO, deferoxamine; LPS, lipopolysaccharide; SE, standard error.

## Discussion

A wide array of health benefits of DFO have been reported in different animal models and human studies [58-60]. In contrast, no studies evaluating the effect of DFO in neuroinflammatory processes induced by LPS administration have been conducted to date. Based on previous studies showing that DFO can protect neurons against degeneration in animal models of brain injury that involves local inflammatory processes, PD and AD [54,61,62], and evidence that sepsis can cause cognitive impairment in human subjects [63,64], we hypothesized that DFO might modify the adverse effects of regional inflammation on cognitive function. Using LPS to elicit an immune response, we observed a significant impairment of memory performance in the MWM test, which was counteracted by DFO.

The MWM was chosen as a robust and reliable test that is strongly correlated with hippocampal-dependent memory [65-67]. As we have stated, both spatial reference memory and working memory were tested [43]. Trial-dependent learning was assessed using a modified protocol. Search-to-platform area determines the degree of reliance on spatial *vs*. non-spatial strategies. Cued trials determine whether performance factors that are unrelated to place learning are present. Escape from water is relatively immune from activity or body mass differences, making it ideal for experimental models. In the preliminary experiment we chose 10 ng, 100 ng, 2 μg, and 5 μg of LPS (intracerebroventricular administration) because these dosages have been reported to induce sickness behavior in mice [68-71], and we sought whether they could impair cognitive function and which one is the applicable and preferred option. We observed no significant differences between groups during the acquisition phase, and 2 μg of LPS (intracerebroventricular administration) caused memory deficits on postoperative day 1 following the microinjection. Mice treated with 2 μg of LPS did not remember where the platform was, and searched aimlessly instead. Both 10 ng and 100 ng may not be sufficient to induce a cognitive decline, but the reason that 5 μg failed to impact behavioral performance remains unknown. We speculate that it triggered some self-protective mechanisms, but this speculation needs future verification. Similarly, we chose 0.5, 1, 2.5, and 5 μg of DFO for blocking LPS-induced behavioral lesions because they were defined to be protective for inflammatory attack, so did the 3 days of time span for pretreatment [71]. We found that 0.5 μg of DFO ameliorated the memory deficits caused by LPS. Likely, we believe that 0.1 μg of DFO was not able to withstand LPS-induced inflammatory cascade, but 2.5 and 5 μg of DFO removed overmuch iron from neurons, hindering iron-indispensable activities like the tricarboxylic acid cycle and thus playing a harmful role [72-74].

In formal experiments, similar results were obtained using 2 μg of LPS and 0.5 μg of DFO. Despite no difference in locomotor activity, DFO prevents memory deficits caused by cerebral administration of inflammatory bacterial LPS both in cue- and trial-dependent learning, which mirrors spatial conference and working memory [43]. In terms of body weight loss, intracerebroventricular LPS induced a sickness response for over 3 days. In the present study, intracerebroventricular LPS induced sickness behavior, as measured by body weight loss and decreased spatial memory. However, these impairments were significantly attenuated by pretreatment with DFO, a high-affinity iron chelator to bind Fe^3+^ [75]. Collectively, our results indicate that LPS causes cognitive impairment, specifically a deficit in short-term memory retention, which can be ameliorated by DFO pretreatment. This finding suggests a potential application of DFO to patients with or at risk for cognitive impairment.

Cognitive decline is prominent in patients who undergo various operations, and also occurs in other disorders in which neuroinflammation is believed to play a prominent role [76]. Thus, we next evaluated the effect of DFO on neuroinflammatory processes induced by LPS administration. Microglia is the resident macrophage in the brain, participates in the coordination of events important for the maintenance of neuronal health, and plays the most important role in responding to inflammation in the central nervous system. Previous studies [8,40,41] have shown that LPS activates microglia and consequently induces pro-inflammatory protein secretion within 6 h via the NF-κB pathway in the mouse hippocampus. Release of these pro-inflammatory cytokines by microglia fuels a cycle of neuroinflammation that can cause bystander damage to neurons [77]. In the current study, we demonstrated that intracerebroventricular injection of LPS induced increases in TNF-α and IL-1β levels in the mouse brain 6 h after the injection and at 24 h postinjection, they returned to the baseline levels. In addition, microglia labeled by Iba1 in the hippocampus was activated. Our results showed that DFO inhibited LPS-induced microglial activation and production of pro-inflammatory mediators including TNF-α and IL-1β. Since that increased iron levels could affect the functional properties of activated microglia at the secretory and gene expression levels and that DFO possesses protective properties against LPS-induced increased iron levels and neural injury, we infer that these effects of DFO are associated with decreased activation of inflammatory cascades linked to pro-inflammatory response [62,71,78].

As reported previously, LPS causes cognitive lesions and this process involves apoptosis [79,80]. Thus, we investigated whether cell death induced by treatment with intracerebroventricular LPS occurred via apoptosis and whether iron was involved. To address this question, caspase-3 activation, an apoptotic marker, was evaluated [81,82]. Caspase-3 as an important executer of apoptosis, was observed to elevate in the LPS-exposed hippocampus and this increase was prevented by DFO pretreatment, from which we could infer that DFO, or the opposite factor iron, may intervene in LPS-induced cell death. On the other hand, GSK3 as a downstream target of Akt regulates many crucial cellular processes in the brain and acts as an important positive regulator of the inflammatory process [83,84]. GSK-3β overexpression induces apoptosis and causes a drastic decrease in postsynaptic density number and volume in hippocampal granule neurons [85], a phenomenon that may be related to cognitive impairment. In this study we observed that intracerebroventricular LPS depressed the phospho-GSK3β/GSK3β ratio in the hippocampus, and dysregulation of this signal transduction pathway could result in failure to adequately repress GSK-3β, thus allowing GSK-3β to remain abnormally active. Such a status is proved to contribute to various pathologies, including neurodegenerative and cognitive disorders, which is in line with the behavioral performance [81,83]. However, DFO alleviated the reduction by phosphorylating GSK3β in the hippocampus and regulated GSK-3β activity in response to LPS, thus providing protection from LPS-induced apoptosis and cognitive impairment. This observation might be caused by the activating effect of Fe on GSK3β [86], which neutralizes the effect of highly activated CDK5 on p-GSK3β suppression. Moreover, DFO treatment highly induced the phosphorylation of GSK3β (inactive form of GSK3β), which in turn downregulated caspase-3 in the LPS-exposed mouse brain.

Since aberrant iron accumulation in the brain is found in many diseases involving cognitive function decline and neuroinflammation plays a pivotal role in many iron-associated neurodegenerative diseases [20,31], we measured the iron content and main proteins involved in the maintenance of brain iron homeostasis in mice to investigate the influence of LPS and DFO on iron metabolism in the hippocampus that is responsible for learning and memory function in the brain. As demonstrated in this study, intracerebroventricular LPS led to a significant increase in iron content (by 66.16%) and DFO dramatically blocked this increase. Accompanied with iron content, Fn accumulation and FPN reduction occurred in the hippocampus of mice in the LPS group, indicating that LPS triggered iron accumulation and export malfunction, which could cause neuronal injury. Peptide hepcidin plays an essential role in maintaining normal iron homeostasis mainly by downregulating FPN, and it can be upregulated by LPS in neuron via the IL-6/STAT3 signaling pathway, as Qian *et al.* demonstrated [87,88], which is in line with our data. Otherwise, DFO as a high-affinity iron chelator to bind Fe^3+^, removes iron from neurons, which is excreted via biliary-fecal and/or urinary routes. In the present study, DFO dramatically blocked iron content increase, reversed Fn accumulation and weakened FPN reduction. This result suggests that there may be causal relationships between LPS-induced neuroinflammation, iron accumulation, and memory deficits, and DFO breaks this tie by diminishing iron in neurons.

Increased levels of brain iron have been proposed to cause neuronal injury via oxidative stress [89,90]. Accordingly, under pathological conditions, iron would catalyze free radical reactions, known as Fenton or Haber-Weiss reactions, causing damage to lipids, DNA, and proteins. In the present study, high levels of iron were found in the mouse hippocampus after intracerebroventricular administration of LPS, suggesting that increased brain iron could be a source of the redox active metals. Indeed, we found significant changes in oxidative stress markers in mice after LPS microinjection. Specifically, MDA in the hippocampus reached about twice that of the control, and SOD activity was reduced. It is tempting to speculate that increased brain iron content leads to the generation of toxic free radicals and the occurrence of oxidative stress, ultimately leading to neuronal injury and cognitive impairment. However, further studies are needed to test this hypothesis.

Collectively, intracerebroventricular microinjection of LPS caused transient upregulation of cytokines (TNF-α and IL-1β), activation of microglia (Iba1), oxidative stress (SOD and MDA), cell death (caspase-3 and GSK3β), and iron accumulation (iron content, Fn and FPN) during the acute phase. Nevertheless, pretreatment with DFO alleviated these lesions and thereby improved LPS-induced cognitive impairment by inhibiting brain iron accumulation. Taken together, we could come to a conclusion that LPS triggers a transient neurocognitive decline in a mouse model that is associated with brain iron accumulation and a series of inflammatory cascade response, which may be caused by the activation of the iron regulation system to some extent; DFO reduces hippocampal inflammation and consequently the pathological progression by blocking iron accumulation in neurons, eventually improving memory performance. DFO may represent a strategy for preventing neuroinflammation-associated cognitive impairment although the exact mechanism needs to be further clarified to complete and validate this intriguing conclusion.

## Abbreviations

aCSF: Artificial cerebrospinal fluid
AD: Alzheimer’s disease
Con: Control
DFO: Deferoxamine
ELISA: Enzyme-linked immunosorbent assay
GSK3β: Glycogen synthase kinase 3β
LPS: Lipopolysaccharide
MDA: Malondialdehyde
MWM: Morris water maze
PD: Parkinson’s disease
POCD: Postoperative cognitive dysfunction
SE: Standard error
SOD: Superoxide dismutase
